# Grading systems in head and neck dysplasia: their prognostic value, weaknesses and utility

**DOI:** 10.1186/1758-3284-1-11

**Published:** 2009-05-11

**Authors:** Stijn Fleskens, Piet Slootweg

**Affiliations:** 1 Department of Otolaryngology/Head and Neck Surgery, University Medical Center St Radboud, PO Box 9101, 6500 HB Nijmegen, the Netherlands; 2 Department of Pathology, Radboud University Nijmegen Medical Centre, PO Box 9101, 6500 HB Nijmegen, the Netherlands

## Abstract

**Background:**

Grading of dysplasia, including head and neck lesions, continues to be a hotly debated subject. It is subjective and lacks intra- and inter-observer reproducibility due to the insufficiency of validated morphological criteria and the biological nature of dysplasia. Moreover, due to the absence of a consensus, several systems are currently employed.

**Objectives:**

The aims of this review are to:

1) Highlight the significance of dysplasia and the importance of a valid method for assessing precursor lesions of the head and neck.

2) Review the different histopathological classification systems for grading intraepithelial lesions of the head and neck.

3) Discuss and review quality requirements for these grading systems.

**Conclusion:**

Regarding the different classification systems, data concerning the WHO classification system are the most available in current literature. There is no simple relationship or overlapping between the classification systems. Further studies should be done to see whether other systems have advantages above the current WHO system and to discover indications that could lead to an universal classification system for intraepithelial lesions of the head and neck.

## Introduction

Head and neck squamous cell carcinoma (HNSCC) is one of the most often encountered malignancies; it carries a bad prognosis.[1,2] To improve survival, adequate diagnosis and treatment of precursor lesions is urgently needed. These precursor lesions are defined as an altered epithelium with an increased likelihood for progression to squamous cell carcinoma (SCC). The altered epithelium shows a variety of cytological and architectural changes that have traditionally brought under the common denominator dysplasia.[3]

The presence of dysplastic areas in the epithelium of the upper aerodigestive tract (UADT) is believed to be associated with a likely progression to cancer. There is evidence that in an individual lesion, the more severe the dysplasia the greater the likelihood is of progression to malignancy. Rarely however, non-dysplastic lesions may also show malignant development .[3-8] Therefore, presence and severity of dysplasia cannot be used as a reliable guide for the treatment of individual cases. Nevertheless, the crude relationship between grading dysplasia and risk of progression to malignancy makes dysplasia grading necessary.

Grading of dysplasia, including head and neck lesions, continues to be a hotly debated subject. It is subjective and lacks intra- and inter-observer reproducibility due to the insufficiency of validated morphological criteria and the biological nature of dysplasia .[8-10] Moreover, due to the absence of a consensus, several systems are currently employed.[11]

Nevertheless conventional histopathological evaluation based on light microscopic examination of hematoxylin & eosin-stained slides is, in spite of the above mentioned shortcomings, still the most valid method for assessing the malignant potential of preneoplastic head and neck lesions.[4] Moreover, it is important to notice that making a diagnosis is a prerequisite for selecting the treatment which ensures the best prognosis, making the disease classification system a predictive system.[8,12] The aim should be to tailor forms of therapy to the likelihood of disease progression thus reducing the incidence of invasive disease, limiting the need for radical surgery and improving survival while avoiding unnecessary follow-up in cases which lack significant premalignant potential.[10]

## Grading systems: overview

During the last decades many classifications of intraepithelial head and neck lesions have been proposed as illustrated by the fact that for intraepithelial laryngeal lesions, more than 20 classification systems have been described .[13-18] This seriously hampers the assessment of the long-term risk of subsequent malignancy, because different histopathological classifications and initial interventions make comparison of reported data difficult or even impossible because of inconsistencies in the criteria used for evaluation of the histological features.[8,19,20] The need for uniformity in reporting these lesions is obvious.

The majority of the classifications in the current literature have followed criteria similar to those in common use for the grading of epithelial lesions of the uterine cervix.[21,22] Whether this is justified, is debatable. In cervical epithelium, there is clear distinction between normal and abnormal layers of the epithelium and consequently the degree of dysplasia can be assessed by determining the horizontal level of this border in the epithelium, resulting in substantial intra- and inter-observer consistency. Such a sharp distinction between normal and abnormal layers in the epithelium of the UADT is less obvious and consequently the definition of the degree of dysplasia is much more susceptible for discussion, this resulting in substantial intra- and inter-observer variability.[8,10,23]

When looking at the current classification systems as mentioned in the WHO-IARC blue book series the following ones are proposed. The WHO classification is similar to the classification for the uterine cervix, and is widely used in spite of the shortcomings as mentioned. It recognizes low, moderate and severe dysplasia and carcinoma in situ (CIS), defined in the same way as for cervical lesions. The SIN classification (squamous intraepithelial neoplasia) can be considered synonymously, excepted that severe dysplasia and CIS are combined as SIN 3 (Table 1). Besides the WHO and SIN classification system, the Ljubljana classification is mentioned (Table 1).[3,11]

**Table 1:** Classification systems that categorize intraepithelial head and neck lesions.[3,11]

**2005 WHO Classification**	**Squamous Intraepithelial Neoplasia (SIN)**	**Ljubljana Classification Squamous Intraepithelial Lesions (SIL)**
squamous cell hyperplasia		squamous cell (simple) hyperplasia

mild dysplasia	SIN 1	basal/parabasal cell hyperplasia*

moderate dysplasia	SIN 2	atypical hyperplasia**

severe dysplasia	SIN 3***	atypical hyperplasia**

carcinoma in-situ	SIN 3***	carcinoma in-situ

* basal/parabasal cell hyperplasia may histologically resemble mild dyplasia, but the former is conceptually benign lesion and the latter the lower grade of precursor lesions.

** 'risky epithelium'. The analogy to moderate and severe dysplasia is approximate.

*** the advocates of SIN combine severe dysplasia and carcinoma in-situ.

The Ljubljana classification, developed by laryngeal pathologists and used since 1971, focuses on the clinical decision points which involve the identification of: 1) purely hyperplastic lesions that do not require close follow-up (simple or abnormal hyperplasia), 2) mild degrees of atypia who require close follow-up to recognize any progression to severe atypia (atypical or 'risky' hyperplasia), 3) severe atypia (carcinoma in situ) who require treatment (surgery or radiotherapy).[4,16,19,24,25]

Apart from these taxonomic problems, the need to differentiate between severe dysplasia and carcinoma in situ is debatable. Several authors have commented on the difficulty in separating these categories in conventional classification systems.[4,5,13] To reduce categories still further, occasionally a binary classifying system has been proposed.[10,26]

## Quality requirements for grading systems

A grading system can be devised in two ways: an arbitrary system can be composed with no detailed knowledge of the domain, or data about the domain can be used in statistical methods of analysis. Since most grading and scoring systems in histopathology are imposed on domains without prior data analysis, the psychological factors that affect the creation of these systems becomes important. There is often an interaction between histopathological grading systems and clinical therapies especially if trials of treatment for a particular condition are widespread. Some authors suggest that the pathologist is required only to divide cases into the number of different treatment options available. Others, including Morris, argue that the pathologist should transmit the maximum amount of information possible from their interpretations without the addition of extraneous 'noise'.[27,28] Furthermore, reproducibility and prognostic value (use of results) are important conditions.

## Reproducibility

Reproducibility refers to the degree to which observer measurement or diagnosis remains the same on repeated independent observations of an unchanged characteristic.[29] This consistency can be assessed between different observers (interobserver) or within a single observer (intraobserver). When studying the accuracy in grading dysplasia of the UADT there is no test available, which is thought to be better than the pathologist's observation; an accepted gold standard is not available for assessing the validity obtained when grading these lesions. Therefore, reproducibility, normally used to assess precision, is used to provide an indication of validity. When combined for this goal, inter- and intraobserver agreement levels give an estimate of the degree of bias and validity in situations (like grading dysplasia of the UADT), where an appropriate gold standard is not available.[30]

If a scoring system is to be clinically useful then it should be reproducible both between pathologists and for the same pathologist at different times: inter- and intra-observer reproducibility.[27] In histopathology, results of the diagnostic evaluation are discrete diagnostic categories (for example, moderate dysplasia in a laryngeal lesion) rather than variable parameters and for this reason kappa statistics are often used as indicators of performance .[31-33] Kappa statistics measure levels of agreement between observers and make allowance for the degree of agreement that would occur by chance alone.[34] Since most grading systems in squamous lesions produce an ordinal categorical result then kappa statistics are a relevant means of assessing reproducibility.[8,27,32,34,35] A kappa statistic of 1 represents perfect agreement and 0 represents the level of agreement expected by chance alone.[27] Landis et al. described guidelines to interpret the quantitative significance of kappa.[31]

Concerning the head and neck region, data of oral lesions outnumber laryngeal lesions. Pindborg et al. for the first time indicated the need for an internationally accepted set of criteria for oral epithelial dysplasia in 1975.[36] Since then, several studies have shown large intra- and interobserver variability in the assessment of intraepithelial head and neck lesions (Table 2).[10,26,30,36-41]

**Table 2:** Observer variability in head and neck lesions.

**Studies/References**	**Localisation**	**Number of slides**	**Histopathological classification**	**Number of examinators**	**Agreement**	**Kappa value**
Abbey et al. 1995	oral cavity/oropharynx	120	WHO**°**	6	35.8–57.5%	0.15–0.41

Fischer et al. 2004^1^	oral cavity/oropharynx	87	WHO**°**	24		0.59 (95% CI: 0.45–0.72) 0.70 (95% CI: 0.56–0.84)^2^

Karabulut et al. 1995	oral cavity/oropharynx	100	WHO**°**	4	49–69%	27–45%^3^

Tabor et al. 2003	oral cavity/oropharynx	43	WHO	3	53%	0.58

Abbey et al. 1998	oral cavity/oropharynx	120	WHO**°**	6	38.5%	0.17^4^

Brothwell et al. 2003	oral cavity/oropharynx	64	WHO**°**	3	51%	0.37

Kujan et al. 2006^1^	oral cavity/oropharynx	68	WHO and binary system ("low-risk" or "high-risk")	4	WHO: 37.7% (unweighted) 92.8% (weighted) Binary system: 74.3%	WHO: 0.22 (95% CI: 0.11–0.35 unweighted) 0.63 (95% CI: 0.42–0.78 weighted) Binary system: 0.50

Mclaren et al. 2000	larynx	100	WHO and two-grade (low and high grade)	13		WHO: 0.32 Two-grade: 0.52

**° **= WHO is not explicitely stated, but terms are in agreement with this system.

^1 ^= The unweighted kappa considers all disagreements to be equally important, while the weighted kappa (Kw) yields a higher reliability when disagreements between raters are small compared with when they are large.

^2 ^= the pathologic diagnoses are restricted to three categories ('no abnormality/hyperkeratosis', 'mild, moderate, or severe dysplasia', 'carcinoma in situ/carcinoma').

^3 ^= when comparing the kappa values between the two pairs of pathologists with the same education, these values did not diverge from the general level of kappa values, indicating that the interobserver variability was due to individual differences rather than to educational background.

^4 ^= Clinical information submitted with biopsy. Same population as [37].

There are also additional features that negatively influence reproducibility. Fischer et al. suggested that inflammation, lesion site, and biopsy technique (punch and wedge) modifies the reliability of oral histological lesions.[38] Clinical information submitted with biopsy specimens did not increase accuracy and consistency.[41]

With these considerations in mind, reproducibility for the larynx gave an overall kappa value of 0.32 for the WHO classification, whereas the use of a two grade system (low and high grade) gave a kappa figure of 0.52.[10] Data concerning the SIN classification and Ljubljana classification in relation to reproducibility of laryngeal lesions are not available in current literature. Agreement for lesions of the oral cavity and oropharynx varies from 35.8 to 92.8% for the WHO classification, kappa values varying from 0.15 to 0.59.[26,30,37-41] A binary system (high/low risk), evaluated by Kujan et al., resulted in 74.3% agreement and a kappa value of 0.50. Particularly for the cases of moderate dysplasia the binary grading system may have merit in helping clinicians to make critical decisions.[26] Fischer et al. also reduced the number of pathologic diagnoses to three categories ('no abnormality/hyperkeratosis', 'mild, moderate, or severe dysplasia', 'carcinoma in situ/carcinoma') which resulted in a kappa value of 0.70 (compared with 0.59 using the various pathologic diagnoses separately).[38] Data concerning the SIN classification and Ljubljana classification in relation to reproducibility of oropharyngeal lesions and lesions of the oral cavity are not available in current literature. Table 2 shows an overview of observer variability in head and neck lesions.

## Prognostic and predictive value

Altmann et al. and Putney et al. reported the first follow-up studies of precancerous conditions of the larynx; carcinoma in situ and keratosis, respectively.[42,43] Since then several follow-up studies concerning the natural evolution and long-term risk of malignant progression in intraepithelial lesions have been reported. In these studies, usually no distinction between natural evolution without treatment (prognostic value) and predictive value (response to treatment) is made. Therefore, data on malignant progression as summarized below concern both treated and untreated cases.

Malignant progression of intraepithelial laryngeal lesions diagnosed with the WHO classification is, according to current literature, as follows: hyperplasia 0–3%.[13,44,45], mild dysplasia 0–30% [13,45-47], moderate dysplasia 0–44% [5,13,45-49], severe dysplasia 20–57% [13,45,47,49,50], CIS 0–80% [13,44,45,48-52].

Regarding the SIN classification, relevant figures are as follows: SIN I 5%, SIN II 25%, and SIN III 11–25%.[5,53] Clinical data concerning follow-up on laryngeal lesions graded with the Ljubljana classification showed a marked increase in the incidence of malignant progression from simple, abnormal, and atypical hyperplasia (resp. 0.7%, 1.0%, and 9.5%).[25] Recently, a study by Gale et al. showed 1.1% (12/1089) progression to carcinoma of squamous hyperplasia/basal-parabasal hyperplasia and 9.5% (17/179) of atypical hyperplasia (CIS is not included).[20]

Few studies have examined the cancer risk related to different grades of oral dysplasias .[54-57] Silverman et al. reported malignant transformation in 36% of cases with oral dysplasia. The degree of dysplasia is not specified.[56] Schepman et al. reported 12% malignant transformation in oral lesions histopathological classified with the WHO classification. Leukoplakias consisting of moderate or severe epithelial dysplasia, had a significantly higher risk of developing a carcinoma than leukoplakias of a lower stage (p < 0.01).[55] In another study 26% of cases with hyperplasia/mild dysplasia and 67% of cases with moderate/severe dysplasia developed into carcinoma.[54] Lumerman et al. studied malignant transformation in hyperplasia with dysplasia, mild dysplasia, moderate dysplasia, severe dysplasia, and CIS; respectively 29%, 8%, 17%, 17%, 0%.[57] Data concerning the SIN classification in relation to predictive value of oral lesions are not available in current literature. The only study in current literature which studied the application of the Ljubljana classification to grading oral intraepithelial lesions has been published by Zerdoner et al. No cases of simple (0/79) or abnormal (0/42) hyperplasia showed progression to carcinoma, 18.2% (2/11) of atypical hyperplasia progressed to invasive cancer.[58]

Interpretation of these reported data of oral intraepithelial lesions are hampered by small sample sizes, surgical intervention carried out for high-risk dysplasias and variability in reporting dysplasia grades. The greater part of published data only considered macroscopical features (i.e. leukoplakia) and no histology.[59,60] It should also be noted that the predictive value of dysplasia is dependent on the prevalence of leukoplakia in a given population.[8] Size, and not histology seems to be the most important in predicting malignant transformation.[61]

So, it appears that regarding reproducibility as well as in terms of prognosis, still a lot of progress has to be made.

Molecular markers are subject of investigation: in spite of many studies, the molecular events that induce the development of premalignancies to carcinoma are still unknown, and we are still forced to conclude that over (or under)-expression of biomarkers itself adds little predictive value over standard histology .[62-66] Therefore, until now they are not applicable in clinical practice.[67]

Finally, some remarks have to be made on the relationship between clinical aspects and risk of malignant progression. In general, homogeneous leukoplakic lesions are thought to have a low risk of malignant transformation, mixed white and red lesions (or speckled leukoplakia) an intermediate risk, and pure erythroplakia (red lesions) the highest risk of cancer development. However, none of these macroscopic features is reliably diagnostic of any histological grade of precursor lesion, and histological analysis of these lesions is mandatory to determine their biological potential. Occasionally precursor lesions may appear clinically normal.[3,11,20,68] Furthermore, nomenclature or terminology concerning the macroscopic features is still a subject of discussion.[69]

## Evaluation

As outlined before, a histological dysplasia system ideally should meet two basic requirements. At first, it should be easily applicable in daily routine practice with low inter- and intra-observer variability. Secondly, it should allow a clear separation between patients who need treatment to prevent progression towards malignancy and those for whom no treatment is needed.

Regarding inter- and intraobserver variability, evaluation of the WHO classification shows for laryngeal lesions an overall kappa value of 0.32, whereas the use of a two grade system (low and high grade) gave a kappa figure of 0.52.[10] Its prognostic significance is as follows: hyperplasia 0–3%.[13,44,45], mild dysplasia 0–30% [13,45-47], moderate dysplasia 0–44% [5,13,45-49], severe dysplasia 20–57% [13,45,47,49,50], CIS 0–80% [13,44,45,48-52]. For oral lesions, inter- and intraobserver figures of the WHO classification vary between kappa scores of 0.15 and 0.59.[26,30,37-41] Its prognostic significance is 12–67%, as can be inferred from the data mentioned before.

When looking at the SIN classification it has to be noted, that with respect to reproducibility, no data of head and neck lesions are available in current literature. Concerning prognostic significance of laryngeal lesions the following data are available: SIN I 5%, SIN II 25%, SIN III 11–25%.[5,53] Data concerning the SIN classification in relation to predictive value of oral lesions are not available in current literature.

Regarding the Ljubljana classification, its use for the larynx has been documented extensively. Its relevance for prognosis has been amply demonstrated by the pathologists and clinicians who developed the system. However, its usefulness has not yet resulted in widespread acceptance. For the oral cavity, there is only one study that reports its use in this anatomic location.[58] In that study a prognostic significance, similar to the larynx was noted. However, data on reproducibility are also lacking for this anatomic area. Further studies should be done to see whether it has an advantage above the current WHO dysplasia system.

**Figure 1:**
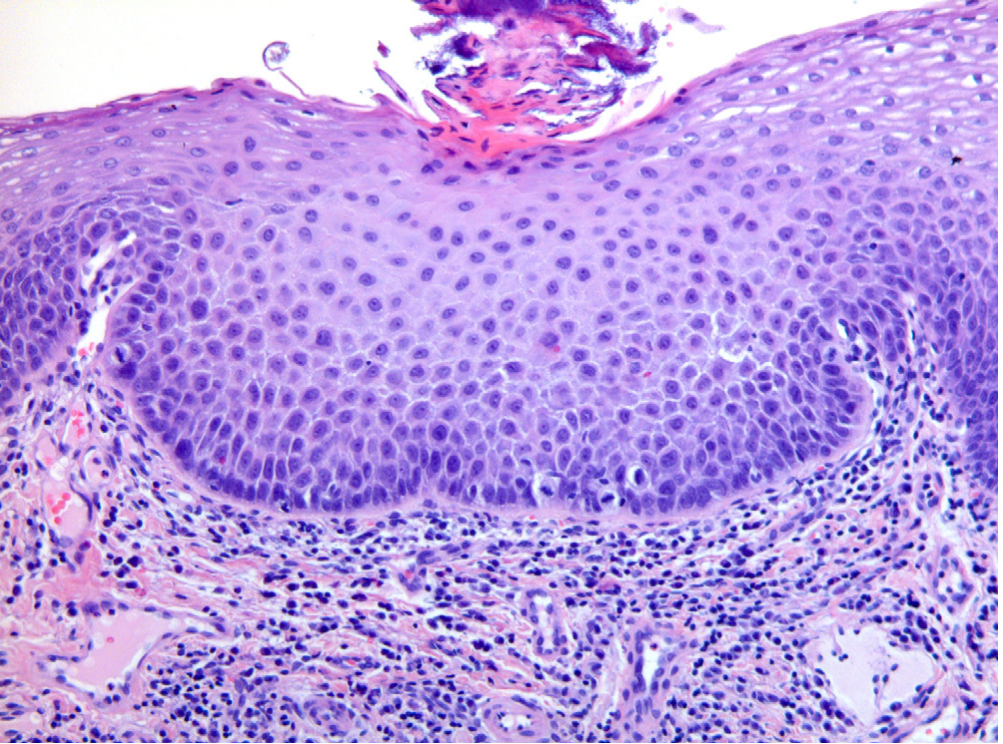
**Photomicrograph showing area of increased epithelial thickness together with hyperkeratosis: mild dysplasia (WHO) or parabasal hyperplasia (Ljubljana)**.

**Figure 2:**
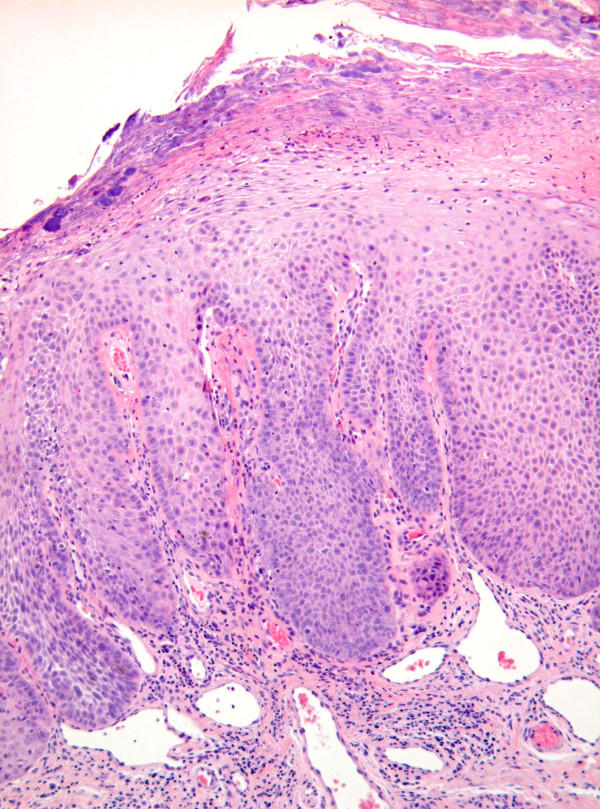
**Photomicrograph showing blunt and elongated epithelial ridges and cytonuclear atypia confined to the lower epithelial half: moderate dysplasia (WHO) or atypical hyperplasia (Ljubljana)**.

**Figure 3:**
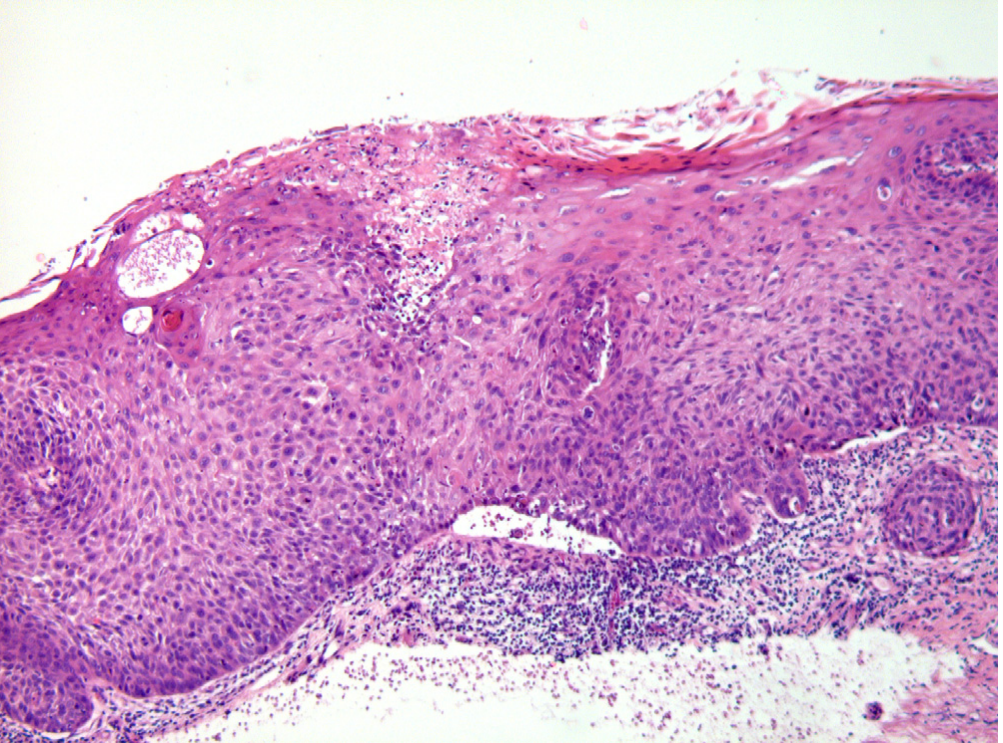
**Photomicrograph showing epithelial alterations involving the entire epithelial thickness: severe dysplasia (WHO) or atypical hyperplasia (Ljubljana)**.

Although the histological assessment of the WHO dysplasia system and the Ljubljana system are based on the same architectural and cytological changes, there is no simple relationship or overlapping between the classification systems.[3,11,19,20] Figure 1, figure 2, and figure 3 illustrate the areas of similarity in the classification systems but also the problems arising when matching the WHO categories moderate and severe dysplasia with the Ljubljana category atypical hyperplasia. According to Gale et al., comparing the three discussed classification systems, it is unlikely that they will come together in the very near future. On the other hand, future discoveries mainly in molecular biology could be the basis for a single, universal classification system for intraepithelial lesions of the UADT.[20]

## Competing interests

The authors declare that they have no competing interests.

## Authors' contributions

All authors read and approved the final manuscript.
